# Supplementary figures and images for: Network Pharmacological Analysis through a Bioinformatics Approach of Novel NSC765600 and NSC765691 Compounds as Potential Inhibitors of CCND1/CDK4/PLK1/CD44 in Cancer Types
Source: Cancers (Basel). 2021 May 21;13(11):2523. doi: 10.3390/cancers13112523 (PMC8196568; doi:10.3390/cancers13112523)

## Slide 1
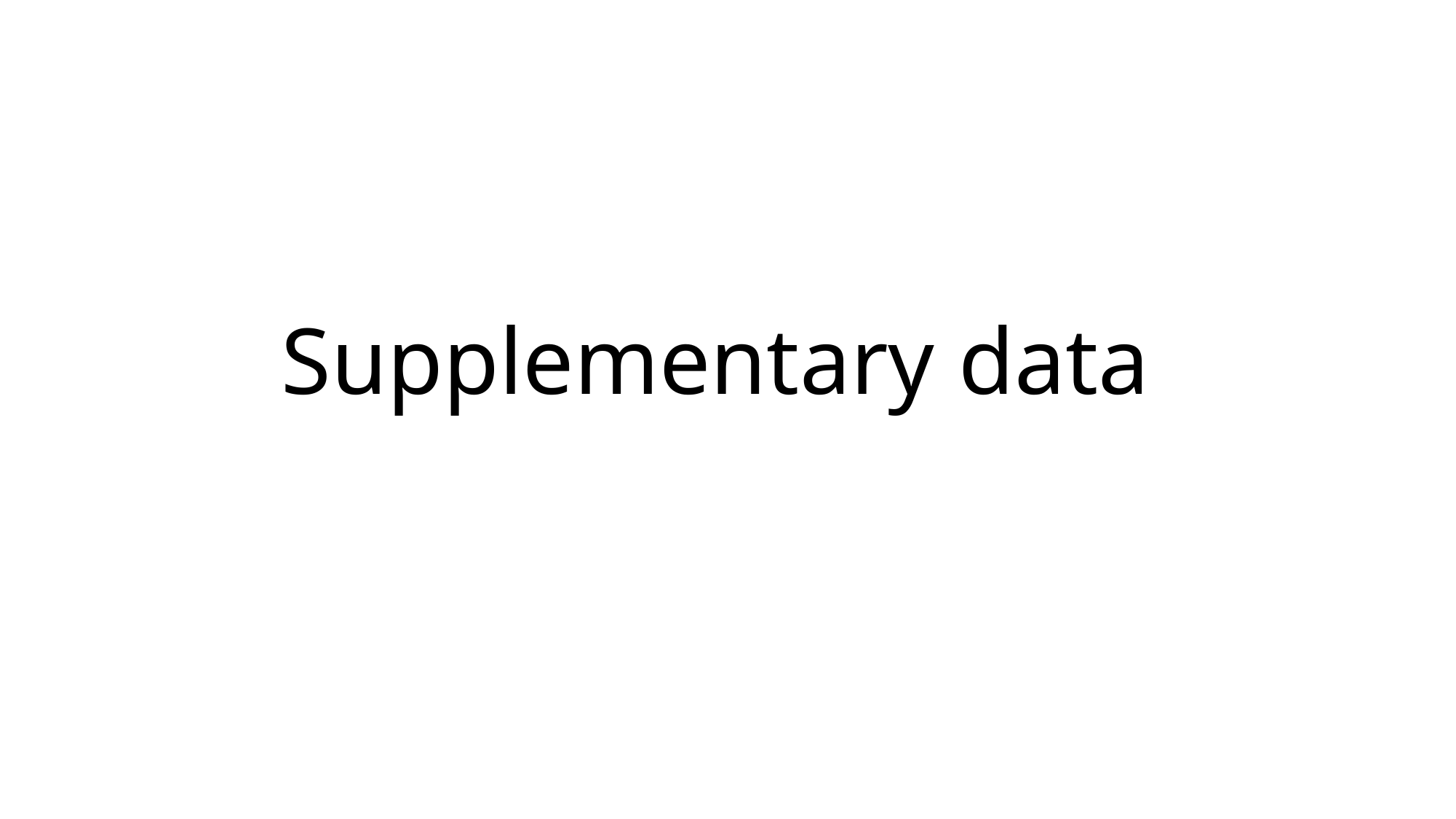

# Supplementary data

## Slide 2
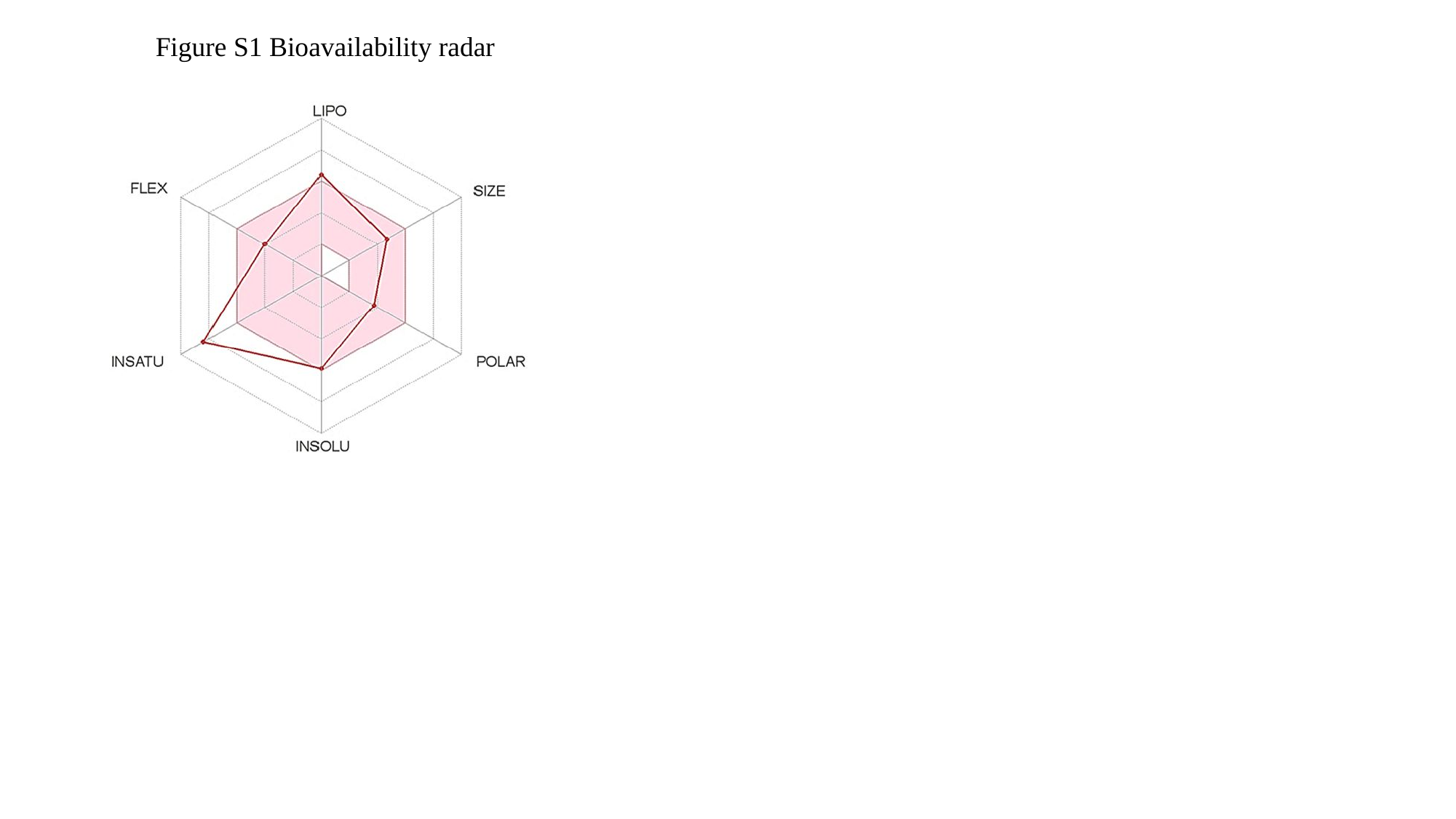

# Figure S1 Bioavailability radar

## Slide 3
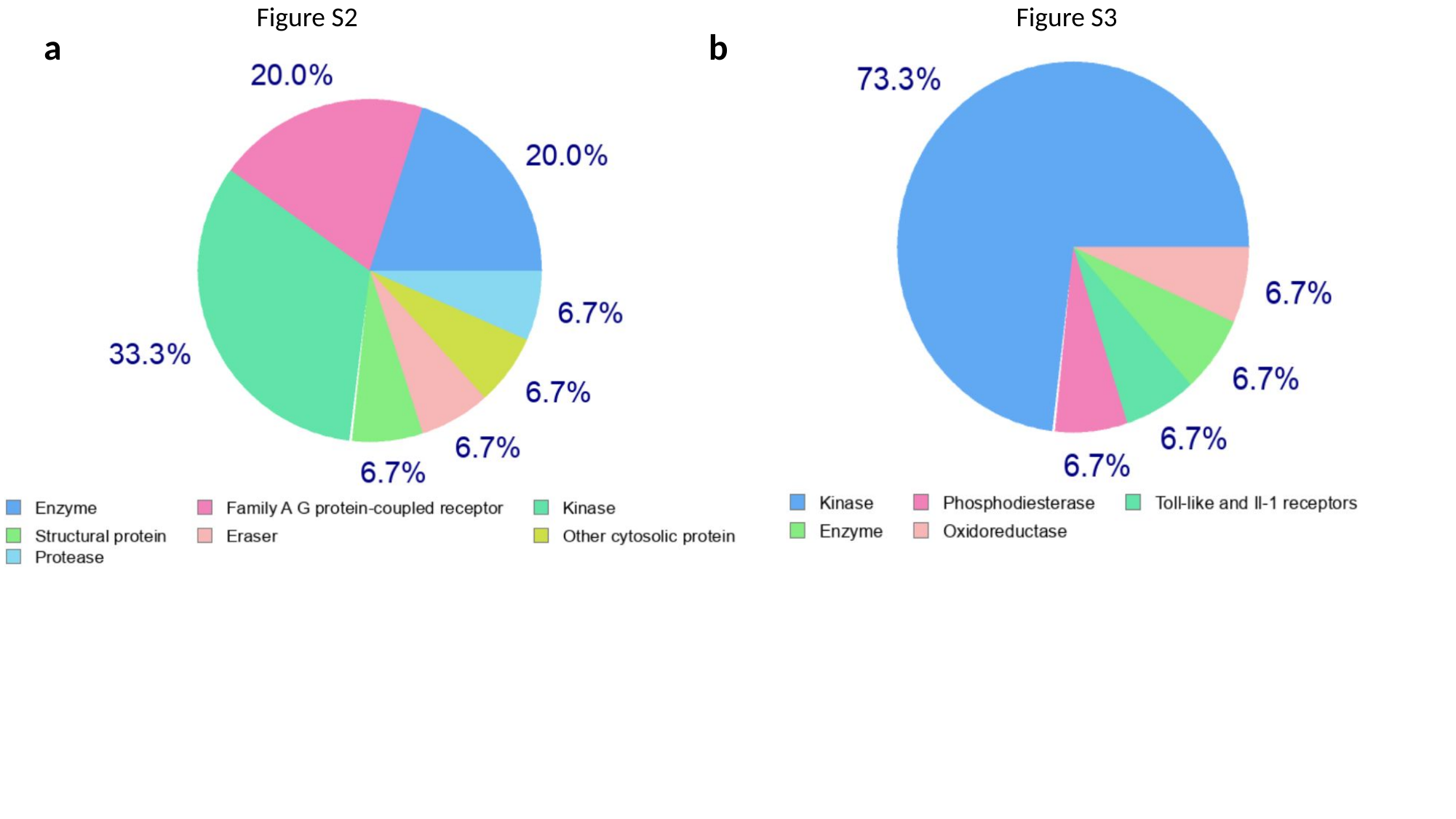

Figure S2
Figure S3
b
a

Supplement: Supplementary file 1 [file cancers-13-02523-s001.zip › Supplementary data.pptx]
